# Ethical considerations for involving adolescents in biomedical HIV prevention research

**DOI:** 10.1186/s12910-021-00695-3

**Published:** 2021-09-23

**Authors:** Rita Nakalega, Carolyne Akello, Brenda Gati, Clemensia Nakabiito, Monica Nolan, Betty Kamira, Juliane Etima, Teopista Nakyanzi, Doreen Kemigisha, Sophie C. Nanziri, Stella Nanyonga, Maria Janine Nambusi, Emmie Mulumba, Florence Biira, Hadijah Kalule Nabunya, Simon Afrika Akasiima, Joselyne Nansimbe, Joel Maena, Juliet Allen Babirye, Kenneth Ngure, Andrew Mujugira

**Affiliations:** 1 Makerere University-Johns Hopkins University (MU-JHU) Research Collaboration, Kampala, Uganda; 2 Department of Global Health, University of Washington, Seattle, USA; 3 Department of Community Health, Jomo Kenyatta University of Agriculture and Technology, Nairobi, Kenya; 4 Infectious Diseases Institute, College of Health Sciences, Makerere University, Kampala, Uganda; 5 Department of Epidemiology and Biostatistics, College of Health Sciences, Makerere University, Kampala, Uganda

**Keywords:** Ethics, Adolescent girls, Biomedical HIV prevention research, Stakeholder engagement, Uganda

## Abstract

**Background:**

Involvement of adolescent girls in biomedical HIV research is essential to better understand efficacy and safety of new prevention interventions in this key population at high risk of HIV infection. However, there are many ethical issues to consider prior to engaging them in pivotal biomedical research. In Uganda, 16–17-year-old adolescents can access sexual and reproductive health services including for HIV or other sexually transmitted infections, contraception, and antenatal care without parental consent. In contrast, participation in HIV prevention research involving investigational new drugs requires adolescents to have parental or guardian consent. Thus, privacy and confidentiality concerns may deter adolescent participation. We describe community perspectives on ethical considerations for involving adolescent girls in the MTN 034 study in Uganda.

**Methods:**

From August 2017 to March 2018, we held five stakeholder engagement meetings in preparation for the MTN 034 study in Kampala, Uganda (NCT03593655): two with 140 community representatives, two with 125 adolescents, and one with 50 adolescents and parents. Discussions were moderated by the study team. Proceedings were documented by notetakers. Summary notes described community perspectives of adolescent participation in HIV research including convergent, divergent or minority views, challenges, and proposed solutions.

**Results:**

Most community members perceived parental or guardian consent as a principal barrier to study participation due to concerns about adolescent disclosure of pre-marital sex, which is a cultural taboo. Of 125 adolescent participants, 119 (95%) feared inadvertent disclosure of sexual activity to their parents. Community stakeholders identified the following critical considerations for ethical involvement of adolescents in HIV biomedical research: (1) involving key stakeholders in recruitment, (2) ensuring confidentiality of sensitive information about adolescent sexual activity, (3) informing adolescents about information to be disclosed to parents or guardians, (4) offering youth friendly services by appropriately trained staff, and (5) partnering with community youth organizations to maximize recruitment and retention.

**Conclusions:**

Stakeholder engagement with diverse community representatives prior to conducting adolescent HIV prevention research is critical to collectively shaping the research agenda, successfully recruiting and retaining adolescents in HIV clinical trials and identifying practical strategies to ensure high ethical standards during trial implementation.

## Introduction

In sub-Saharan Africa, adolescent girls and young women (AGYW) accounted for 25% of HIV infections in 2020, despite comprising 10% of the population [1]. In this setting, six in seven new HIV infections among 15–19 year old adolescents occur among girls [1]. The susceptibility of adolescent girls to HIV infection is increased by social, cultural, biologic, and economic factors including age-disparate sexual relationships, transactional sex, intimate partner violence, inability to negotiate condom use, genital inflammation, and cervical immaturity [2–6]. To protect and promote the health of adolescent girls and eliminate HIV transmission, the World Health Organization (WHO) recommends offering biomedical HIV prevention interventions, including oral pre-exposure prophylaxis (PrEP), to this vulnerable population as part of combination prevention [7]. Thus, the inclusion of adolescent girls in HIV prevention trials is essential to better understand the efficacy and safety of new biomedical HIV prevention interventions, including injectable PrEP, topical PrEP (intravaginal rings, gels, or films), implants and vaccines [8]. There is also need for researchers to further understand adolescent perspectives about the acceptability, adherence, and preferences for investigational HIV prevention products [9]. Nonetheless, there are significant ethical and regulatory challenges to enrolling adolescents into clinical trials, including myths and misconceptions, parental/guardian consent, distrust of the research process, limited access to clinical trials, and stigma related to participation in research [9, 8, 10]. Consequently, adolescents remain under-represented in biomedical HIV prevention trials [11, 12] despite their inclusion being important [13].

In the United States, Department of Health and Human Services guidelines (45 CFR part 46, subpart D) describe procedures for inclusion of adolescents in a clinical trial, outline levels of risk that a research may pose to a child, and permit only three categories of research to be carried out among children [14]. These are: (1) research not involving greater than minimal risk; (2) research involving greater than minimal risk but presenting the prospect of direct benefit to the individual subjects; and (3) research involving greater than minimal risk and no prospect of direct benefit to individual subjects, but likely to yield generalizable knowledge about the subject’s disorder or condition [14]. Research Ethics Committees (REC) in Uganda have adopted this clinical trial standard and require it to be met prior to approval of research to be conducted in children. Adolescent HIV prevention research is included under category 2 and is thus ethically permissible [9, 13].

In Uganda, girls aged 16–17 years old can access sexual and reproductive health (SRH) services, including HIV and other sexually transmitted infections (STI), contraception, and pregnancy care on their own [15, 16]. However, research on investigational new drugs (IND) requires adolescents to provide consent in addition to obtaining permission from a parent or guardian [17]. Parental/guardian involvement may also lead to inadvertent and unwanted disclosure of adolescent sexual activity thus compromising privacy and confidentiality, and undermining trust and engagement in research [8, 18]. Parental involvement hinders recruitment of adolescents into HIV prevention research because of cultural taboos about sex education and pre-marital sex in sub-Saharan Africa [10]. Stakeholder engagement with diverse community partners is necessary to ensure that clinical trials are collectively designed and implemented in mutually beneficial ways [19]. Good Participatory Practice (GPP) guidelines recommend stakeholder engagement throughout the various stages of biomedical HIV prevention trials to assure ethical design and conduct of research [20]. Engagement of key stakeholders ensures that there is ‘buy-in’ at multiple levels and serves as a platform to identify address cultural and social barriers and facilitators to adolescent HIV prevention research [20]. We describe stakeholder perspectives of ethical considerations for research involving adolescent girls participating in the Microbicide Trials Network (MTN) 034 study in Uganda.

## Methods

### Setting

This study was conducted at Makerere University—Johns Hopkins University (MUJHU) Research Collaboration in Kampala, Uganda, one of the four participating sites in the MTN-034 study. MTN-034, is an ongoing Phase 2a, multi-site, randomized, open-label, crossover study that enrolled 247 healthy, HIV-negative adolescent and young adult females aged 16–21 years [ClinicalTrials.gov NCT03593655]. The study period was 72 weeks total, divided into three 24-week segments. During weeks 1–48, participants received two different biomedical HIV prevention products, each of which was administered in a sequence, for a total of 24 weeks. The interventions were: (1) an intravaginal ring containing 25 mg of dapivirine, dispensed, and replaced monthly, or (2) one 200 mg FTC/300 mg TDF oral tablet, dispensed monthly with counseling to take daily. The sequence of interventions was randomly assigned. In the last 24 weeks of the study, participants were offered the choice of continuing with either product, or no product at all.

### Data collection

Between August 2017 and March 2018, we conducted five consultative meetings with key stakeholders before recruiting AGYW into the MTN-034 study. Two stakeholder meetings were held with AGYW aged 16 to 21 years purposively selected by the adult and youth community advisory board (CAB) to represent diverse views and age groups. A total of 125 AGYW willing to discuss HIV prevention methods attended the meetings, including those in school, out of school, and breastfeeding. A joint meeting with adolescents and their parents or guardians was attended by 50 females. Two stakeholder engagement sessions were held with 140 adult participants including local council leaders, youth organization heads, community youth leaders, medical professionals, women representatives and leaders from policymakers, regulatory bodies, and ethics review committees, the National Drug Authority (NDA), Joint Clinical Research Centre (JCRC), Ministry of Health, WHO, and Uganda National Council for Science and Technology (UNCST). We included individuals from diverse professional backgrounds who had different roles in AGYW health care provision to obtain a wide range of perspectives.

Stakeholder meetings aimed to create awareness about the MTN-034 (REACH) study, gain community insights about the study, and learn about community perspectives of engaging adolescents younger than 18 years (the legal age of consent) in sexual reproductive health [SRH]-related research. The agenda and specific objectives of each meeting were discussed in advance by the study team. Specific objectives included: (1) to provide an overview of the oral PrEP and vaginal ring landscape in Uganda; (2) to explain the design of the REACH study; (3) to seek views on contraception requirements for enrollment and to discuss the contraceptive method mix that would be offered in REACH, especially long-acting reversible contraceptives such as intrauterine devices and implants; and (4) to identify strategies to disseminate information to parents and guardians and facilitate enrollment of young women into the study. Female research staff gave presentations to meeting attendees about each objective. Thereafter, discussion sessions were led by two to three moderators using guides based on each of the presented topics. The meeting held with regulators and community stakeholders focused on reviewing UNCST guidelines regarding participation of emancipated minors in research. These guidelines define emancipated minors as individuals below the age of majority (i.e., < 18 years), who are pregnant, married, have a child, or cater for their own livelihood. They stipulate that emancipated minors can independently provide informed consent to participate in research if: (a) in the view of the IRB the research is not objectionable to parents or guardians, and (b) the research protocol includes clear justifications for targeting mature and emancipated minors as participants, and for not involving parents or guardians in the consent process. Key stakeholders were probed to elicit their views about parental consent, waiver of consent, and parental role in the engagement of young women in research. AGYW opinions about sex, sexuality, and SHR including MTN-034 participation were explored in adolescent-only sessions.

Notes were taken during each meeting by study team members who were not moderating the sessions. Proportions of AGYW reporting sexual activity and contraceptive use were documented. Proceedings of stakeholder meetings were reviewed by the study team and summarized. The team presented summary results that were relevant to ethical considerations for engaging adolescents in an HIV prevention trial as discussed by the different stakeholders.

This study was approved by the JCRC REC, UNCST (HS2314), NDA (CTA0044), and Johns Hopkins Medicine Institutional Review Board (JHMIRB IRB00154539). Informed consent was obtained from all participating stakeholders including legally authorized guardians or parents of AGYW.

## Results

Below, we highlight key stakeholder perspectives as documented in summary notes.

### National and community stakeholders

Regulators and community stakeholders strongly supported conducting the MTN-034 study in Uganda. After reviewing UNCST guidelines, there were varying opinions about waiver of parental consent for emancipated minors. Some stakeholders thought that there was no need for parental consent even though it was an Investigational New Drug (IND) trial because the guidelines permitted waiver of parental consent. However, waiving parental consent was culturally unacceptable to others. Overall, parental consent was perceived as a barrier to meaningful participation in adolescent research in a setting where parents play a key role in the health decisions of their daughters. Thus, there was need to provide more clarity in the guidelines regarding waiver of parental consent for IND trials. After further discussion with the study team, community members agreed that it was essential to obtain parental or guardian consent before engaging adolescents younger than 18 years in IND HIV prevention research.

Stakeholders recommended that high-risk adolescents, including emancipated minors, should be targeted for enrollment because they were sexually active and would benefit from HIV prevention services available in the study. They reasoned that since adolescent HIV prevention research was a relatively new concept in Uganda, studies should recruit emancipated minors, and AGYW known to be sexually active, before including other adolescents. Some stakeholders contended that school going adolescents should not be recruited because this could be misconstrued as promoting promiscuity and hinder study retention. Stakeholders also feared that clinical trial participation could interfere with school attendance. A female local council leader expressed concern that including AGYW in the study appeared to be encouragement for them to start having sex. Furthermore, exposure to sexually experienced emancipated peers was perceived to be a negative influence.

Stakeholders also emphasized the need for high ethical standards during study implementation including maintaining confidentiality, respecting privacy and autonomy, and protecting adolescents from harm since this vulnerable population is easily exploited. They maintained that if these issues were not well handled, they could hinder adolescent participation in research Community stakeholders nominated representatives to serve on a social harms committee that would be constituted to address any issues arising from participation in research. Community members agreed to work with the study team to support education of parents/guardians, increase awareness about HIV prevention, and facilitate adolescent participation in HIV trials.

### Parents and guardians

The regulatory requirement for parental or guardian consent was perceived as a principal barrier to adolescent study participation by all parents and guardians. Their key concern was that disclosure of pre-marital sexual activity was considered a cultural taboo. Some parents narrated how sex-related issues were never discussed with them when they were growing up because sex education was frowned upon in their culture. Subsequently, they too avoided discussing sex with their children.

Parents and guardians misunderstood their role in the study beyond providing informed consent. They thought that they would have to be present at each clinic visit and be involved in all counseling sessions with their daughters. Parents and guardians were also concerned that participation in HIV research could result in adolescent stigmatization through being perceived as HIV-positive or as “high risk” children (i.e., sexually active or substance users). They feared that this could have a negative impact on the social reputation of the family and lead to community discrimination or exclusion. In response, the study team acknowledged that HIV-related stigma was one of the risks of involvement in research. They described the strategies employed to minimize risk, including keeping participant information confidential, and having a dedicated adolescent clinic located separately from adult clinics at the research site to ensure privacy.

#### Adolescents

AGYW felt that the study would benefit all who were sexually active. However, 95% (119/125) of them feared unwanted disclosure of their sexual activity to parents or guardians. Some adolescents disclosed that they were sexually active and felt that they could make their own decisions about study participation. Most 16–17 year-old girls felt that they did not need parental or guardian consent to participate in research. One girl quipped that she did not see the need to involve her parents in consenting to participate in research because she did not seek their consent before having sexual relationships. Additionally, 30% (38/125) of adolescents reported that they were already accessing SRH services, including contraception, without parental or guardian consent. Another girl said she didn’t see the need for parental consent because she already accessed family planning services of her own volition. One-in-four (31/125) adolescents reported that they did not live with their parents or guardians. Some were orphans while others had traveled to the city for work and were staying alone or with relatives. Thus, they felt that the need for parental or guardian consent would hinder their research participation.

## Discussion

Stakeholder consultative meetings held prior to MTN-034 trial implementation improved study conduct in multiple ways. Transparent and mutually respectful stakeholder engagement with diverse community representation was critical to gaining a richer understanding of community perspectives and concerns about female adolescent participation in HIV research. Stakeholder engagement helped to improve research literacy, and empower and equip stakeholders to meaningfully engage in increasing awareness about oral PrEP and vaginal ring studies conducted at the research site. The study team harnessed community stakeholder expertise to identify ethical considerations for adolescent research in the local setting, and to facilitate study recruitment and retention. These stakeholder meetings for adolescent HIV prevention research were the first ever held in the country.

Stakeholders highlighted the need for community endorsement of research studies. Community endorsement for the MTN 034 study was expressed through stakeholder acknowledgment of adolescent HIV risk, support of prevention research and importance of the study. Community organizations pledged to dispel myths and misconceptions about adolescent HIV prevention research and to support the recruitment of study participants. Community engagement is key to preventing alienation of communities when research studies are being planned and implemented [21]. Additionally, community input is needed during study design to identify social and cultural perspectives that may interfere with the conduct of a research study. Collaborative partnerships with adolescents, parents and communities may also enhance study recruitment and retention [10].

Whereas protection of adolescents and minors is prudent, and indeed necessary, largely complex and conflicting legal and ethical regulations—or a lack of guidance about their operationalization, as is the case in most low- and middle-income counties—have caused a reluctance to conduct adolescent-centric research [5, 22]. There has been much debate about parental consent for participation in research by minors. Emancipated minors are permitted to provide consent for research that doesn’t involve investigational new drugs (IND) in Uganda [17]. On a fundamental level, parental consent is vital especially since research shows that adolescents are more likely to make decisions impulsively and to overestimate benefits while underestimating risks [23]. Adolescents may not be able to fully appreciate the long-term implications of research participation and thus may require parental consent which provides an additional layer protection for informed and ethical participation of adolescents in biomedical research [24]. Despite all these, the onus is on researchers to make all efforts to boost adolescent comprehension of research [25]. Additionally, protection of adolescents from harm in research needs to be judiciously paralleled with the need to carry out studies in this demographic. Rigid ethical guidelines and legislation, especially regarding the requirement for parental consent, may constrain adolescent participation in HIV prevention trials [9], especially since sexually active adolescents enrolled into biomedical studies are recruited from HIV, perinatal and family planning clinics. The sexual activity of “mature minors” is frowned upon by parents and communities and considered taboo [18]. Waiving parental consent on the other hand may not always be culturally acceptable or may act as a barrier to adolescent participation in research since parental involvement in adolescent healthcare is normative in this setting.

We found that a quarter of adolescents were living alone either because their parents were deceased or lived in distant rural communities. Others lived under the care of relatives who were not formally recognized as the child’s legal guardians. Obtaining parental consent for such adolescents is difficult as described in similar settings in South Africa and Nigeria [10, 18, 26] Current ethics guidelines may not reflect the reality and logistical complexities inherent in obtaining consent from absentee parents or guardians, or the complex privacy and confidentiality concerns of adolescents. To encourage adolescent participation in IND clinical trials, ethics committees should provide clear guidance regarding waiver of parental or guardian consent in circumstances where it is warranted. The reality of mature minors and adolescent-headed households as a result of the HIV pandemic in Uganda [27] needs to be recognized because such adolescents are able to provide independent informed consent. This could improve uptake and use of biomedical interventions, study adherence and retention, and community acceptance of adolescent participation in studies that could have groundbreaking public health implications [18].

To successfully include female adolescents in HIV research in Uganda, we recommend good participatory practices that effectively engage young women, parents and guardians, community organizations and civil society and regulators using a combined “bottom up” and “top down” approach. Fostering collaborative and mutually beneficial stakeholder partnerships enables researchers to gain an informed understanding of socio-cultural traditions, respect norms and practices regarding adolescent sexuality, address priorities and concerns of parents and guardians, and listen to the voices of young women as equal partners. Gaining the trust of key stakeholders and ensuring “buy in” for IND trials is essential for successfully conducting adolescent HIV research.

This study has limitations. We did not perform a formal qualitative study; stakeholder meetings were not audio recorded, qualitative discussion guides were not used, and meeting proceedings were not subjected to qualitative data analysis. Thus, direct quotes and themes are not provided. Nevertheless, our findings inform engagement of a critical demographic in HIV research. Stakeholder meetings were conducted in Kampala, the capital city of Uganda, where stakeholders were likely to have heard of, or been exposed, to AGYW research. However, some key stakeholders were from national organizations with an urban and rural footprint thus providing generalizable views. Policies regarding adolescent informed consent vary by country limiting the generalizability of our findings. Nevertheless, parental concern about adolescent sexuality and research participation is nearly universal.

## Conclusions

High HIV incidence among adolescent girls in sub-Saharan Africa supports their inclusion in HIV prevention research. Adolescents should not be denied the benefits of research in keeping with the ethical principle of justice. Respect for adolescent autonomy must be maintained throughout the informed consent process and participation in research. Despite the ethical challenges posed by adolescent participation in HIV prevention research, collaborative partnerships with community stakeholders can help to successfully recruit and retain adolescents in clinical trials.

## Data Availability

The summary notes obtained and analyzed during this study is available from the corresponding author upon request.

## Abbreviations

AGYW: Adolescents and young women
HIV: Human immunodeficiency virus
MTN: Microbicide trials network
NDA: National drug authority
PrEP: Pre-exposure prophylaxis
SRH: Sexual and reproductive health
STI: Sexually transmitted infections
WHO: World Health Organization
